# Benchmarking organic mixed conductors for transistors

**DOI:** 10.1038/s41467-017-01812-w

**Published:** 2017-11-24

**Authors:** Sahika Inal, George G. Malliaras, Jonathan Rivnay

**Affiliations:** 1 Biological and Environmental Sciences and Engineering Division, King Abdullah University of Science and Technology (KAUST), Thuwal, 23955-6900 Saudi Arabia; 2 Department of Bioelectronics, Ecole Nationale Supérieure des Mines, CMP-EMSE, MOC, 13541 Gardanne, France; 3 Department of Biomedical Engineering, Northwestern University, Evanston, IL 60208 USA; 4 Simpson Querrey Institute for BioNanotechnology, Northwestern University, Chicago, IL 60611 USA; 5 Present Address: Electrical Engineering Division, Department of Engineering, University of Cambridge, 9 JJ Thomson Avenue, Cambridge, CB3 0FA UK

## Abstract

Organic mixed conductors have garnered significant attention in applications from bioelectronics to energy storage/generation. Their implementation in organic transistors has led to enhanced biosensing, neuromorphic function, and specialized circuits. While a narrow class of conducting polymers continues to excel in these new applications, materials design efforts have accelerated as researchers target new functionality, processability, and improved performance/stability. Materials for organic electrochemical transistors (OECTs) require both efficient electronic transport and facile ion injection in order to sustain high capacity. In this work, we show that the product of the electronic mobility and volumetric charge storage capacity (µC*) is the materials/system figure of merit; we use this framework to benchmark and compare the steady-state OECT performance of ten previously reported materials. This product can be independently verified and decoupled to guide materials design and processing. OECTs can therefore be used as a tool for understanding and designing new organic mixed conductors.

## Abstract (web summary)

Organic materials that support both electronic and ionic transport hold promise for applications in bioelectronics and energy storage. Here, Inal et al. use transistors to quantify the materials performance of organic mixed conductors in terms of the product of charge mobility and volumetric capacitance.

## Introduction

Organic mixed conductors are soft, often polymeric materials based on traditional organic electronic materials. These materials support electronic charge transport along their conjugated backbones, while allowing for ionic (mass) transport through the bulk. Like their inorganic counterparts, it is the interaction of ionic and electronic species that allow for the diverse applications ranging from battery electrodes to electrochromic windows and sensors^1^. The interplay between ionic and electronic carriers is critical for energy storage and generation including pseudo/supercapacitors^2, 3^, batteries^4^, fuel cells^5^ and ionic-organic ratchets^6^. Furthermore, organic mixed conductors have found utility in applications where large area processing is required (i.e., electrochromics), where the motion of ions through the bulk can lead to significant changes in physical properties (mechanical actuators, electrochromics, switchable surfaces)^7–9^, or where the material’s intimate contact with a sensing environment can lead to enhanced sensitivity or selectivity (sensors)^10, 11^. Where one application requires selectivity, another requires fast response; where one requires stability, another requires capacity. While device-based characterization of such properties enables comparison, lacking is a materials-based figure of merit to benchmark and guide synthetic/processing design and development.

Organic electrochemical transistors (OECTs) have received significant attention for their promise as circuit elements^12^, neuromorphic devices^13^, and sensing/stimulation elements useful in bioelectronics applications^13–15^. The ability for organic mixed conductors to support ion penetration (and thus bulk charge capacity) has allowed for high performance OECTs to be developed as amplifying transducers for bioelectronic applications^15^. In many cases, the enhanced sensing capability of OECTs has allowed them to outpace both mixed conductor-coated electrodes and similarly sized inorganic transistors based on non-mixed conductors^16, 17^.

**Fig. 1:**
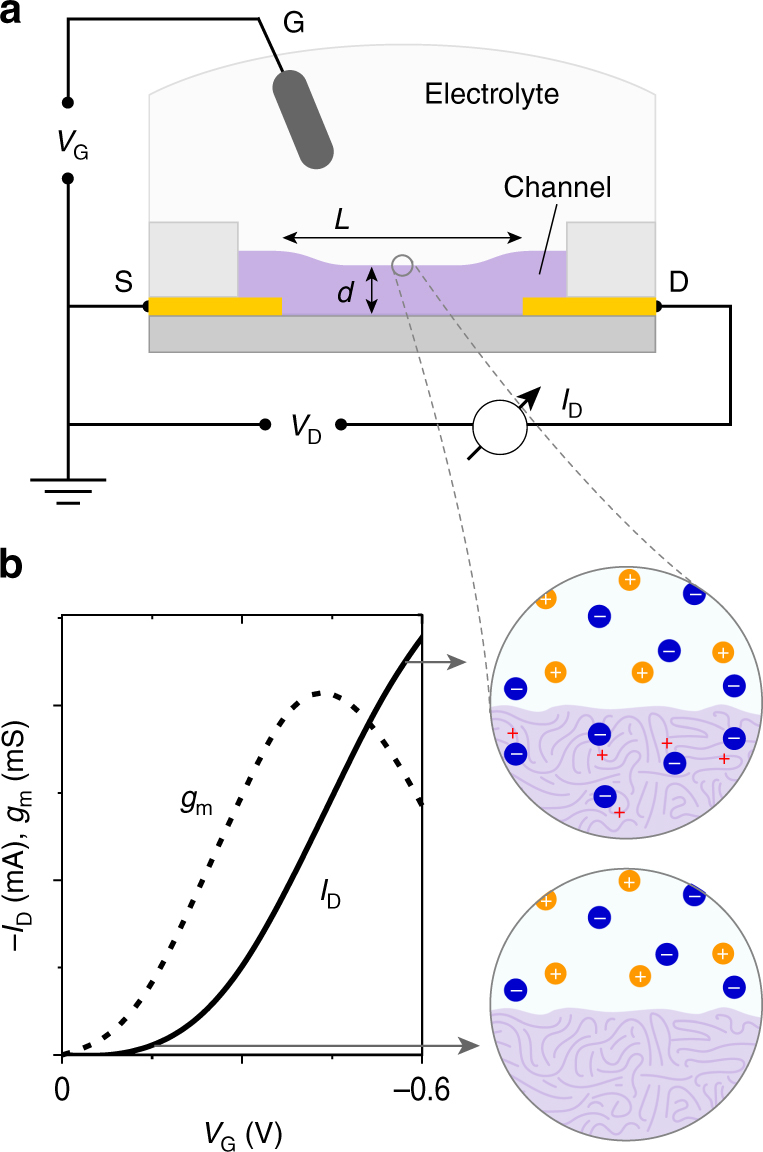
OECT description and operation. **a** OECT cross section, wiring, and dimensions: channel length (*L*), and thickness (*d*). The source, drain, and gate (S, D, G, respectively), and relevant voltages (gate, drain voltage: *V*_G_, *V*_D_) and currents (drain current, *I*_D_) terms are also labeled. **b** Representative transfer (*I*_D_ *−* *V*_G_) curve for a p-type accumulation mode device (*V*_D_ < 0 V), and the corresponding transconductance curve (*g*_m_). The schematics on the right indicate the doping state of the film, where the “ON” state allows for anion drift/penetration and subsequent stabilization of holes on the semiconducting backbone. In the schematics, cations are orange, anions are blue, and holes are red

In an OECT, the mixed conductor channel bridging a source and drain electrode is in direct contact with an electrolyte rich in mobile ions (Fig. 1). The current between source and drain electrodes, *I*_D_, is modulated by a change in effective gate bias, *V*_*G*_ (applied at an immersed gate electrode or resulting from cellular or (bio)molecular activity), through a bulk gating effect facilitated by ion penetration. Operation through bulk doping yields both low bias operation, a hallmark of electrolyte gated transistors, but also high capacitance. Due to the motion of both electronic and ionic charges in the channel, mobility extraction cannot be, in a straight forward manner, performed via the transconductance method as applied for field effect transistors (FETs). For this reason, OECT researchers often report device transconductance, which is the slope of the *I*_D_ *−* *V*_G_ transfer curve, *g*_m_ = ∂*I*_D_/∂*V*_G_ (see Fig. 1b). While this term is sometimes reported as a normalized value (e.g., by channel width, *W*, or by drain voltage, *V*_D_), reporting standards are not well established. Nevertheless, *g*_*m*_ is a direct measure of effective signal amplification of a single OECT; which, for instance, determines whether the OECT will be able to operate as a biosensor, i.e., transduce small biological signals. Furthermore, *g*_m_ depends on both the channel geometry and biasing conditions and describes the steady-state performance of the device. As such, it does not represent the performance of the mixed conductor, yet can be used to derive an appropriate figure of merit for fair materials comparison.

The conducting polymer poly(3,4-ethylenedioxythiophene) complexed with poly(styrene sulfonate) (PEDOT:PSS) is among the most commonly studied materials in OECT research, and has allowed for significant device physics and modeling development. It is stable in aqueous conditions, processable by a variety of methods, allows for both electronic and ionic transport, and importantly, is readily commercially available^3, 18^. Previous work has shown that volumetric capacitance (~40 F cm^−3^ in PEDOT:PSS) governs high transconductance; consequently, the physical film thickness of the OECT channel, *d*, plays a critical role in determining both transient and steady-state device characteristics^15^. These findings can be corroborated and derived^15^, resulting in the following scaling of OECT transconductance:

1 $$g_{\mathrm{m}} = \frac{Wd}{L}\,\mu C^{\mathrm{*}}\left( {V_{{\mathrm{th}}} - V_{\mathrm{G}}} \right)$$

Where *µ* is the electronic carrier mobility, and C* is the capacitance of the channel per unit volume. *V*_th_ is the threshold voltage. In the limiting case where the channel thickness drops to a monolayer, the product of *d* and C* is *C*′ (capacitance per unit area), which collapses to the transconductance equation for FETs:

2 $$g_{\mathrm{m}} = \frac{W}{L}\mu\,C^{\prime}\left( {V_{{\mathrm{th}}} - V_{\mathrm{G}}} \right)$$

In the equation governing the OECT performance (Eq. (1)), the geometry dependence is captured by the *Wd*/*L* term and the gate bias offset by (*V*_th_ − *V*_G_). This leaves the product of *µ* and C* as the only nominally geometry- and bias-independent terms, and thus a materials-system dependent product.

Device-based figures of merit that are treated as materials properties are especially useful in materials evaluation and development. For example, OFET materials are often benchmarked based on their *µ*_FET_, while thermoelectric materials are defined with their *ZT* figure of merit. It is proposed herein that the µC* product is the materials-system figure of merit for OECTs. This term captures the steady-state ionic/electronic transport processes within the channel material, under device operation. Herein, we use this framework to compare and benchmark ten previously published organic mixed conductor materials. We further explore how a decoupling of µC* into *µ* and C* can aid in materials design, including synthetic strategies and processing approaches. Knowing the *µ* and C* of the channel material, we can predict the steady-state performance, i.e., *g*_m_, of an OECT given a certain geometry and operation condition. We suggest that the µC* product could be useful for informing materials selection in other mixed conductor applications.

## Results

### µC* for benchmarking OECT materials

µC* captures the mixed ionic-electronic transport properties of the OECT channel material. When determined using Eq. (1), *µ*, the electronic mobility, reflects the device-scale electronic charge transport in the channel, and has been shown to be comparable to values determined by complementary methods, such as Hall effect mobility^19^. C* is a measure of the capacity of the bulk material (a volumetric term, as opposed to gravimetric, often used for energy storage devices). This term is a convolution of both the ionic penetration/transport within the polymeric morphology, and the ability for the conjugated backbone to store and collect electronic charges. Ultimately, C* should be a steady-state characteristic, and should reach this value at experimentally or application relevant time scales.

**Fig. 2:**
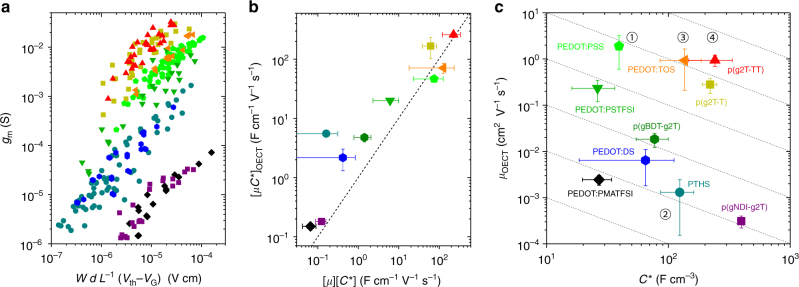
µC* as the OECT figure of merit. **a** Transconductance (*g*_m_) of OECTs as a function operating conditions (in saturation regime) and channel geometry. Each point represents one OECT measurement; each color/shape represent one material, as indicated in the labeling in **c**. **b** The linear slope of the data in **a**, [µC*]_OECT_, as a function of the product of *µ*_OECT_ and C* determined independently, [*µ*][C*]. The dashed line represents 1:1 agreement between the values. Error bars represent the propogation of errors from the product of *µ* and C* for which the standard deviation is determined over all devices tested for each material, as well as the error in the linear fit of data from each material in **a**. The color coding and shape for each material is as noted in **c**. **c** *µ*_OECT_−C* map of ten previously reported materials. Dotted lines denote constant µC* product. Circled numbers in **c** refer to the discussion in text

To extract the µC* product, it is sufficient to measure *g*_m_ and carefully note the device geometry parameters and biasing conditions. However, a more rigorous investigation, where multiple devices of varying geometries are measured, allows for both proper verification of OECT scaling (i.e., *g*_m_~*Wd*/*L*), and for a more statistically supported µC* determination. Following Eq. (1), the data can be plotted as *g*_m_ *vs. Wd* *L* ^*−1*^|*V*_th_ *−* *V*_G_|, where the slope is [µC*]_OECT_. This is shown for the materials investigated in Fig. 2a. Variability or spreading in values can be attributed to non-uniformities in film formation, edge effects and their amplified effects in small devices, and difficulty in measuring thickness for small devices. This µC* product is then plotted for all ten materials in Fig. 2b, where a clear ranking of materials is evident.

Decoupling these terms can provide both validation and lead to a facile route to understand why one material outperforms another. For these materials, *µ* is determined by extracting the electronic carrier transit time (*τ*_*e*_) through the constant gate current^20^, or impedance matching methods previously reported^21^ and described in Methods. C* is measured independently by performing electrochemical impedance spectroscopy (EIS) measurements on a polymer coated microelectrode. The EIS results can be, in all materials discussed here, fit to a simple equivalent circuit: *R*_s_ *C* or *R*_s_(*R*_p_||*C*) where *R*_p_ and *C* are the parallel resistance and the capacitance associated with the polymer, respectively, while *R*_s_ is the sum of the electrolyte and interconnect resistances (Supplementary Fig. 1). The capacitive term in the circuit is then normalized by the product of the measured electrode area and film thickness, i.e., the volume of the film. It is required that a material operates as a true OECT: at reported biases, ion penetration should progress through the bulk of the film (in this simplistic case, lateral ion transport under the applied drain bias is presently neglected)—meaning no stratification, and no dominance from ion accumulation at the electrolyte/channel interface (otherwise accurate C* extraction is non-trivial, and the OECT operation dictated by Eq. (1) does not apply).

The product of these independently measured values [*µ*] and [C*] agree well with the [µC*] product attained from geometry-dependent transconductance plot shown in Fig. 2a (Fig. 2b, Table 1). While many materials do not show a direct 1:1 match and often trend towards an underestimation in the independently derived product [*µ*][C*], the relative relation amongst different materials is maintained. The conjugated polyelectrolyte, PTHS, shows the most significant deviation. With this general agreement in trends, we can properly benchmark materials for OECTs. For example, the prototypical PEDOT:PSS exhibits a µC* of 47 ± 6 F cm^−1^ V^−1^ s^−1^. The top performer to date is p(g2T-TT), a glycolated thiophene-thienothiophene polymer^22^, with a µC* of 261 ± 29 F cm^−1^ V^−1^ s^−1^. It is important to note that in all of the materials shown in Fig. 2, testing was performed with the same setup, using a Ag/AgCl pellet as the reference/gate electrode and 0.1 M NaCl as the electrolyte.

**Table 1:** Materials figure of merit for various OECT materials

Material/Formulation	C* (F cm^−3^)	*µ*_OECT_ (cm^2^ V^−1^ s^−1^)	[*µ*_OECT_][C*] (F cm^−1^ V^−1^ s^−1^)	[µC*]_OECT_ (F cm^−1^ V^−1^ s^−1^)
p(g2T-TT)^32^	241 ± 94	0.94 ± 0.25	227 ± 107	261 ± 29
p(g2T-T)^22^	220 ± 30	0.28 ± 0.1	62 ± 24	167 ± 65
PEDOT:TOS [VPP]^33^	136 ± 50	0.93 ± 0.72	126 ± 108	72 ± 14
PEDOT:PSS + EG^19^	39 ± 3	1.9 ± 1.3	75 ± 51	47 ± 6
PEDOT:PSTFSILi100^24^	26 ± 10	0.23 ± 0.11	6.1 ± 3.8	20 ± 1.6
PTHS + EG^23^	124 ± 38	0.0013 ± 0.0011	0.16 ± 0.15	5.5 ± 0.1
p(gBDT-g2T)^22^	77 ± 23	0.018 ± 0.006	1.4 ± 0.6	4.8 ± 0.7
PEDOT:DS + EG^34^	65 ± 46	0.0064 ± 0.0046	0.42 ± 0.4	2.2 ± 0.9
p(gNDI-g2T)^35^	397	0.00031 ± 0.00009	0.12	0.18 ± 0.01
PEDOT:PMATFSILi80^24^	27 ± 7	0.0024 ± 0.0006	0.06 ± 0.02	0.15 ± 0.01

p(g2T-T), p(g2T-TT) and p(gBDT-g2T) are glycolated thiophene, thiophene-thienothiophene and BDT-thiophene based polymers, respectively. TOS, PSS, PSTFSILi100 and PMATFSILi80, and DS, are various molecular, polymeric, and biological anionic dopants complexed with PEDOT, namely tosylate, poly(styrene sulfonate), (trifluoromethylsulfonyl)sulfonylimide (styrenic or methacrylic backbone, with a molar mass 100 or 80 kDa and Li^+^ as the counter ion), and dextran sulfate, respectively. p(gNDI-g2T) is a glycolated naphthalene diimide based polymer, which is the only n-type semiconductor in this work. PTHS is a thiophene-based conjugated polyelectrolyte with sulfonate terminated alkyl chains. Ethylene glycol (EG) is a co-solvent additive known to improve electronic conductivity of polymer films

### *µ*-C* maps

By plotting the decoupled terms, *µ*-C* maps can be assembled to aide in materials understanding and synthetic design. On such a map, the highest µC* product is at the top right (Fig. 2c), with lines of constant µC* denoted as the dotted diagonal lines. PEDOT:PSS, labeled as ① in Fig. 2c, has long been the top performing material. PTHS ②, a conjugated polyelectrolyte, was thought to be a  promising candidate material due to its tethered ionic groups^23^, however the low µC* product suggests otherwise. By decoupling these terms, it is clear that while this material attains a C* value three times higher than that of PEDOT:PSS, its hole mobility is ~×1000 lower. Thus, to further improve the performance of OECTs based on this material, focus should be placed on enhancing its mobility without hurting C*. On the other hand, PEDOT:TOS ③, a vapor phase polymerized (VPP) PEDOT doped with tosylate (TOS) ions, shows a similarly high C* as PTHS, but maintains a mobility of ~1 cm^2^ V^−1^ s^−1^. p(g2T-TT) ④, the top performing OECT material in this data set, further pushes C* to 241 F cm^−3^, while still maintaining a high *µ*. It is interesting to note that the only n-type material in this set, p(gNDI-g2T), shows the highest C* value (397 F cm^−3^), and yet its mobility keeps it from attaining the high transconductance of its p-type counterparts^22^.

In this dataset, it is clear that *µ* varies over a larger range than C*. However, a number of trends are deduced from Fig. 2c: materials with comparable *µ* result in higher µC* products and thus higher transconductance due to a higher C*. Grouping materials, those possessing excess and bulky ionic or polyionic dopants, i.e. PEDOT doped with various polyanions^24^, show lower C* (<100 F cm^−3^) compared to the rest of the materials. These materials may allow for efficient swelling and faster ion drift and diffusion, however, the “dead volume” from the excess, electrically insulating phase lowers C*. Strategies for enhancing C* are thus important, but doing so while maintaining high mobilities can present a tradeoff that must be deftly navigated^19^.

### Trajectories

**Fig. 3:**
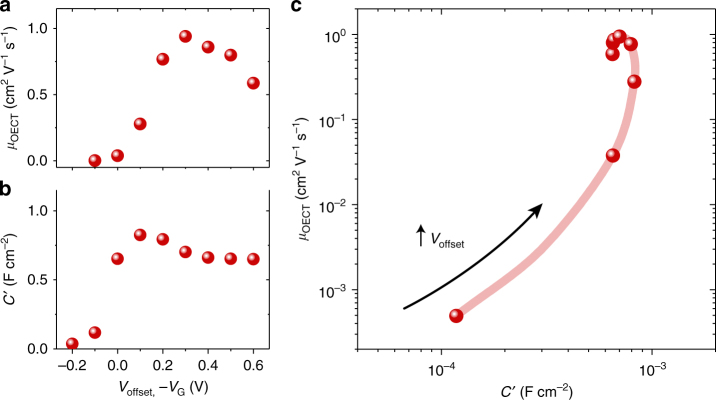
Gate or offset bias trajectory for the p(g2T-TT). OECT mobility **a** and C′ (capacitance per unit area) **b** as a function of bias (*V*_offset_ of working electrode for *C*′, −*V*_G_ for *μ*). **c** *µ-C*′ map of data from **a**, **b** showing an example of a bias trajectory

Nevertheless, distilling a material to a single point in *µ*-C* space can be misleading. For example, while often a mobility value is assigned to an organic electronic material, it is well known that mobility depends on charge density^25^, and can be anisotropic, depending on morphology^26, 27^. Similarly, it has been observed in electrolyte gated organic transistors that as charge density is swept through a broad range (by sweeping the applied gate voltage), both mobility and the observed capacitance can vary significantly, often passing through a peak value^28, 29^. For mobility, this may be attributed to band filling or in some cases, contact effects; for capacitance, the cause is potentially related to barriers for ionic/electronic charge injection. It should be carefully noted that the derivation of Eq. (1) (ideal OECT operation) assumes no such barriers. Thus, care must be taken in data interpretation when operating in regimes of significant voltage dependent capacitance, as noted below. Voltage dependence of *µ* and C* is shown in Fig. 3 for an accumulation mode glycolated polymer, p(g2T-TT). The electronic mobility, determined from an OECT, increases more than three orders of magnitude, with a peak value at 0.3 V (Fig. 3a). When biased over the same range, a drastic increase in capacitance starting at *V*_offset_ ~ −0.1 V is noted, roughly saturating in the 0–0.6 V range (Fig. 3b). Such bias-dependent functions can be represented in *µ-C* space as in Fig. 3c—note that *C*′ is reported rather than C* since the degree of uniformity of ion penetration through the bulk is unknown at low *V*_G_. As mentioned, the applicability of the figure of merit does not necessarily hold over the entire range. The highest transconductance for this device is achieved at the top right of this trajectory which would correspond to the largest µC* product; in this case at *V*_offset_ ~ 0.25 V, where proper OECT scaling is observed. All reported single µ-C* values (Fig. 2) are at peak transconductance in saturation regime, which, in some cases, is the peak transconductance achieved before sample degradation or instability.

**Fig. 4:**
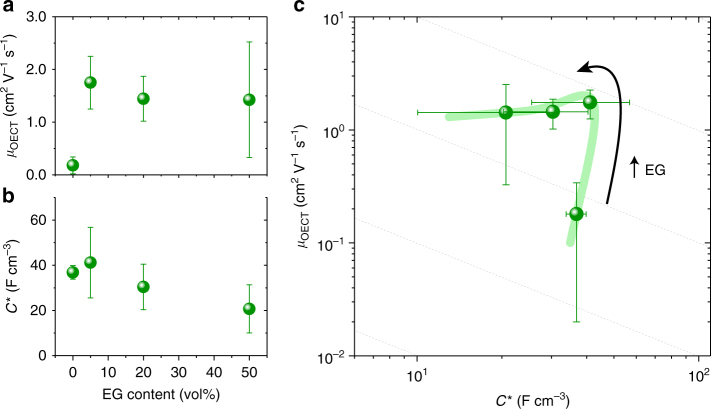
Formulation/morphology trajectory for PEDOT:PSS. OECT mobility **a** and C* **b** as a function of ethylene glycol (EG) concentration. **c** µ-C* map of data from **a**, **b** showing an example of a trajectory corresponding to variations in PEDOT:PSS formulation, and therefore the film morphology/microstructure. Dotted gray lines denote constant µC* product. Samples are the same as those from ref. ^19^. Error bars represent standard deviations from measurements of four transistors

We can envisage other trajectories that can inform a processing or formulation arc maximizing mixed conductivity. An example of this type of trajectory is one where additives or solvent treatments are applied in order to tune the microstructure of the material, and therefore its properties. For instance, ethylene glycol (EG) is a co-solvent commonly used to enhance electronic conductivity of PEDOT:PSS. Recently, we explored how this additive simultaneously affects ion transport^19, 30^. This same formulation set (with EG) can be examined with respect to *µ* and C* (Fig. 4a, b). The µ-C* mapping of this trajectory (Fig. 4c) is in further agreement with the results in ref. ^19^, showing that a peak transconductance occurs at an intermediate EG concentration.

## Discussion

Aside from such processing conditions, the peak µC* product of a material can depend on other factors. Temperature, for example, is an obvious variable. Well controlled experiments with liquid (aqueous) electrolytes are, however, complicated when fine temperature control is desired, limiting the range of accessible temperatures as the morphology of the channel material and the phase and ion concentration of the electrolyte can simultaneously change. A resulting implication is that the µC* product does not depend solely on the dry material properties of the channel. It should also be a system property encompassing the nature of the electrolyte. The size, charge, and nature of the (solvated) ions would have differing ion mobilities^31^, and different interactions with the solid state film; in some cases a large ion may not be able to access the electroactive volume, which may limit C*. In the extreme case, excessively large ions would not be able to penetrate the channel, resulting in a true “field effect” condition, where accumulation would be limited to double layer formation at the channel-electrolyte interface. The solvent of the electrolyte (in the case of liquid electrolytes) could also interact and modify the channel morphology depending on the nature of both polymer and solvent—i.e., hydration and swelling—which could affect both mobility and C*.

It is noteworthy that while the µC* product governs the steady state characteristics of an OECT, it holds little relevance to the transient behavior for materials that show true OECT operation. In fact, *g*_m_ does not include information directly associated with the time scale of (de)charging of the film. The response time of the OECT will be a function of how fast the ions penetrate inside the polymer and the electrons/holes are extracted at the source/drain contacts. While much is known about electronic charge transport, the drift of ions inside the film along multiple directions and how this contributes to the switching speed of the device are challenging to quantify. However, for the material to be volumetrically doped/dedoped within the channel of an OECT on application-relevant time scales, we postulate that there should not be a significant barrier for ion injection and that the mobility of ions must be sufficiently high to allow for complete (de)charging of the (semi)conductor channel. While materials such as PEDOT:PSS show an agreement between the device response time and the *RC* charging time of the transistor channel^15^, this is likely not true for all materials herein. Nevertheless, even the polymers for which ion transport is considered quite slow or at least orders of magnitude slower than PEDOT:PSS (e.g., p(g2T-TT), the top performer) are able to show OECT behavior and fit within the steady state µC* framework.

While ultimately, the µC* product can depend on a number of factors, it is a powerful tool to benchmark materials and can be used to directly compare materials being used as the active channel in OECTs. We have shown that of nearly a dozen reported materials for OECTs, representing the synthetic and materials development work of six academic groups, the magnitude of the µC* figure of merit varies over three orders of magnitude. The deconvolution of electronic mobility and the volume density of bulk charge storage (indicative of ion penetration/transport) aides in materials design, and can thus be used to discuss organic mixed conductors in a common framework. While directly applicable for studying OECT performance from the transconductance perspective, relative materials benchmarking can be a powerful tool to compare organic mixed conductors of interest in other application areas.

## Methods

### OECT characterization

Materials and devices were prepared, as previously reported^19, 22–24, 32–35^. They were all tested as noted in Fig. 1, with a Ag/AgCl electrode (Warner Instruments) immersed in 0.1 M NaCl in deionized water. OECT characterization was performed on either Keithley 2400 source measure units (SMU) or a National Instruments PXIe system (equipped with NI-PXI-4071 digital multimeters, NI-PXI-4143 SMU and a NI-PXI-6289 DAQ), using custom LabView software. Mobility (*µ*_OECT_) was determined on the OECT channels the same or comparable to those used to measure the current–voltage characteristics and to extract the transconductance of the OECTs (i.e., same film casting consitions, comparable dimentions, and same effective gate biasing). Measurements were performed by using the constant gate current method, where drain current transients (d*I*_D_/d*t*) are extracted for different constant gate currents, *I*_G_, at a particular drain bias, *V*_D_. The hole or electron transit time can then be calculated from d*I*_D_/d*t* = −*I*_G_ */τ*_*e*_^17, 20^. Using the same relation, a frequency dependent approach can be employed to extract the hole/electron transit time. A constant drain bias and a sinusoidal voltage signal (Δ*V*_G_ = 10–20 mV) at a gate voltage offset (*V*_G,offset_) are applied depending on the biasing conditions of peak transconductance (similar to EIS experiments). The resulting gate current and drain current sinusoids are extracted to determine both amplitudes and phase shifts. The frequency domain relation derived from the time domain analysis is: Δ*I*_G_(*f*) = 2*πf τ*_*e*_ Δ*I*_D_. Thus, matching the gate current and drain current derived impedance yields *τ*_*e*_, as detailed in ref. ^21^. Using the hole/electron transit time in the channel (*τ*_*e*_), the channel length, and the applied drain bias, *µ*_OECT_ can be estimated.

### Electrochemical impedance spectroscopy

Electrochemical impedance spectroscopy (EIS) for determination of capacitance was measured on polymer coated electrodes using either a Metrohm Autolab PGSTAT128N or Gamry Reference 600 from 20 kHz to 1 Hz. The impedance spectra of the channel of the OECT or a micron-scale film coated on gold electrodes (3.48E-3 cm^2^ in area) were measured in 0.1 M NaCl aqueous solution, using a standard Ag/AgCl as the reference electrode and a Pt mesh as the counter electrode. The measurements were performed at a DC offset potential which enables the maximum achievable doping for the material and an AC amplitude of 10 mV. Once the spectra were recorded, they were fit to an equivalent circuit using native tool software (Metrohm Autolab NOVA or Gamry software) or MATLAB. The simplest circuit with a pure capacitor was used (i.e., *R*_s_ *C*), or Randle’s circuit, *R*_s_(*R*_p_||*C*), and resulted in good fit quality as shown in Supplementary Fig. 1. The capacitance values that are extracted were normalized by the measured film volume or plotted as a function of volume and then fit to a line to determine volumetric capacitance (C*). Thickness of films was measured in the dry state with a Bruker Dektac profilometer.

### Data availability

The data that support the findings of this study are available within the article, its Supplementary Information or from the authors.

## Electronic supplementary material

Supplementary Information
