# Supplementary material for: Benchmarking organic mixed conductors for transistors
Source: Nat Commun. 2017 Nov 24;8:1767. doi: 10.1038/s41467-017-01812-w (PMC5701155; doi:10.1038/s41467-017-01812-w)
Supplement: Supplementary file 1 — Supplementary Information [file 41467_2017_1812_MOESM1_ESM.pdf]

Supplementary Figures

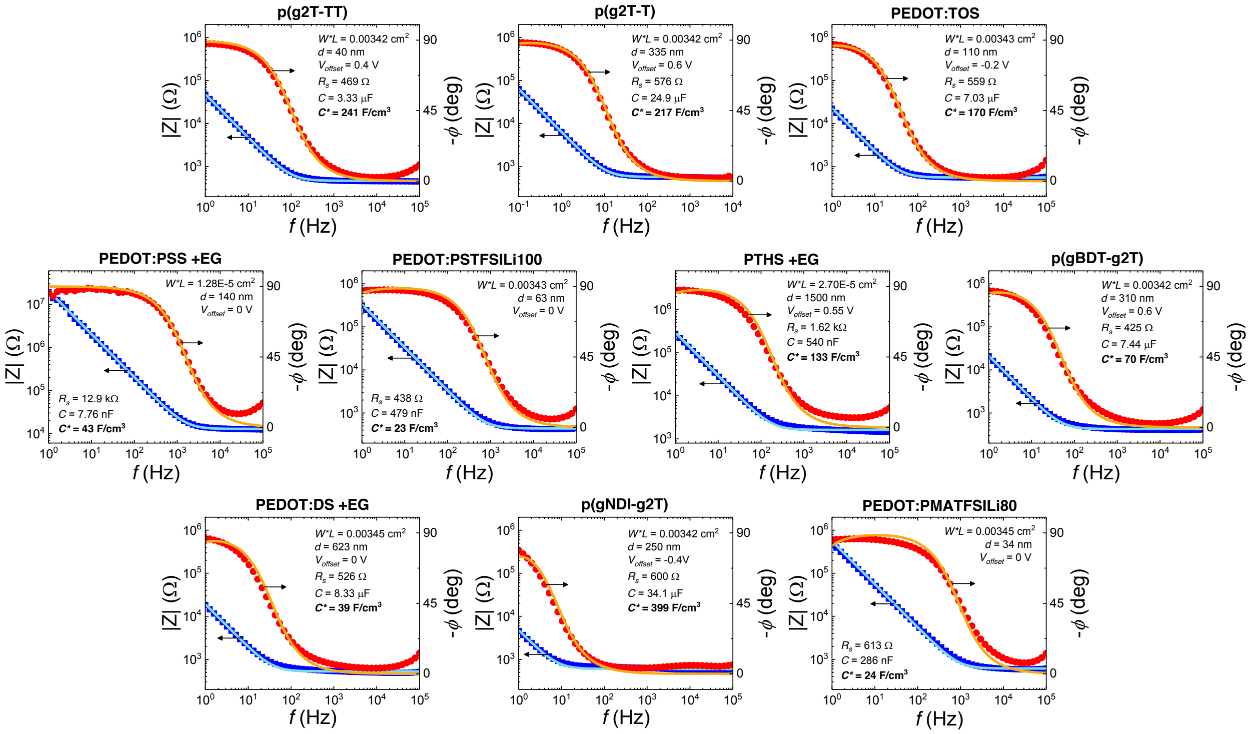

Supplementary Figure 1. Electrochemical impedance spectroscopy (Bode plots) for each material discussed in this work. As these samples are from different sets of original experiments, the specific size or thickness may not be comparable, however, the fits are representative of the data set. For each plot, the modulus of the impedance, $|Z|$ (blue symbols), and phase (red symbols) are plotted as a function of frequency. Fits are shown as lines in light colors ($|Z|$ fit, cyan; phase fit, orange). The results are fit to either an R_sC, or $R_s(R_p||C)$ equivalent circuit where R_s is the electrolyte resistance, R_p and C describe the resistance and capacitance of the polymer film, respectively. In all cases shown, the value of C and R_s have little dependence on which circuit is chosen. The area and thickness, offset bias (V_{offset}), and R_s, C fit results are shown, as well as the resulting C^* value for the particular device.
